# Perceptions of health misinformation on social media: patterns and predictors

**DOI:** 10.3389/fmed.2026.1770275

**Published:** 2026-05-15

**Authors:** Navreet K. Singh, Aisha T. Langford

**Affiliations:** Department of Family Medicine and Public Health Sciences, Wayne State University School of Medicine, Detroit, MI, United States

**Keywords:** false, health information, misinformation, misleading, perception, social media

## Abstract

**Purpose:**

Determine factors associated with perceived amount of health misinformation when using social media.

**Methods:**

Secondary analysis of the Health Information National Trends Survey 7 (HINTS 7). US adults 18 years and older completed surveys in 2024 (*N* = 7,278). Participants with complete data for all variables were included for analysis (*N* = 4,741). Univariable and multivariable logistic regression models were used to identify correlates of interest for how much health misinformation participants saw when using social media. “A lot” and “some” responses were compared to “a little” and “none.” As a sensitivity analysis, “a lot” was compared to all other responses combined.

**Results:**

Higher odds of perceiving “a lot/some” misinformation were observed among adults aged 50–64 years and 65–74 years (vs. 18–34 years), college graduates (vs. non-college graduates), and incomes of $50,000–$99,999 and $100,000 or more (vs. $0–$19,999). Lower odds were identified in Black and Hispanic (vs. White) participants and those who agreed (vs. disagreed) their social media network had the same views on health. Odds of perceiving “a lot” of misinformation were higher in adults aged 50–64 years and college graduates. Lower odds were found in those who: were Black and Hispanic, agreed their social media network had similar health views, had higher trust in the healthcare system, and disagreed (vs. neutral) to a strong sense of ethnic group belonging.

**Conclusion:**

Perceived health misinformation on social media varies by sociodemographic characteristics, trust in the healthcare system, and alignment of health views within social media networks.

## Introduction

In July 2021, during the COVID-19 pandemic, the US Surgeon General’s Advisory issued a public statement to draw attention to health misinformation and provided advice on how individuals and organizations could reduce the spread of misinformation (1). Health misinformation was described as “information that is false, inaccurate, or misleading according to the best available evidence at the time” (1). Social media has been a prominent channel through which misinformation about COVID-19 and COVID-19 vaccination has circulated, and is a common way health misinformation spreads (2–7). For example, Stephan et al. (8) found that most TikTok videos about female pelvic floor conditions were of poor informational quality and that 18 percent contained misinformation. Kaya et al.’s (9) evaluation of YouTube videos on hypertension management determined that nearly half (49 percent) were misleading. Dhanoya et al. (10) reported that among fertility-related content on Twitter/X and Instagram, 74 percent of posts did not cite sources or academic references and 45 percent contained inaccurate information. Loeb et al. (11) examined 150 widely viewed videos on YouTube about prostate cancer screening and treatment, and found that 115 (77 percent) contained biased or poor-quality information. These patterns raise concern given the widespread use of social media among US adults. According to a 2025 Pew Research Center survey, 84 percent of US adults reported using YouTube and 71 percent reported using Facebook, with substantial use of Instagram (50 percent) and TikTok (37 percent) (12).

Evaluating how individuals perceive health misinformation on social media is essential for identifying vulnerable populations, understanding how belief systems shape interpretation and trust, and informing interventions that strengthen the public’s ability to recognize misleading information. A systematic review by Wang et al. (13) emphasized the importance of examining how susceptibility to health misinformation varies across sociodemographic groups and how belief systems contribute to its spread. Prior research suggests that perceptions of information credibility are shaped by a combination of individual characteristics, trust in institutions, information-processing capacity, and social context (13, 14). From an *information processing* perspective, individuals may rely on both analytic and heuristic processes when evaluating health information (15–17). These dual-processing modes have distinct implications: analytic processing involves systematic and effortful thinking, whereas heuristic processing relies on mental shortcuts and peripheral cues. In complex or unpredictable communication environments such as social media, heuristic cues such as trust in the source of information or consistency with prior beliefs may shape judgments of credibility and increase susceptibility to misinformation (15).

Individuals’ positions within the social structure, including education, income, and race/ethnicity, may influence access to and evaluation of health information, as described by *communication inequality* frameworks (18, 19). Trust in the healthcare system is a particularly important factor, as it may influence how individuals interpret health information and which sources they rely on for high-quality health information. Lower trust has been associated with greater skepticism toward authoritative information and increased susceptibility to misinformation (20, 21). Moreover, individuals may be more likely to accept health information that aligns with their beliefs, values, and identities rather than evidence that is factual or objectively derived, consistent with *motivated reasoning* (22). Social media environments can reinforce existing beliefs through exposure to like-minded networks and algorithm-driven content, which may contribute to confirmation bias and differences in perceptions of misinformation (23–25). In parallel, individuals’ capacity to find, understand, evaluate, and use health information, often conceptualized as health literacy or digital health literacy, affects their ability to distinguish credible information from misleading content (26–29).

The present study is a secondary analysis of the 2024 Health Information National Trends Survey (HINTS 7) data. Informed by concepts from information processing theory, communication inequality frameworks, and motivated reasoning, we selected variables identified in the literature as being associated with health misinformation, including: (1) sociodemographic characteristics and health-related factors; (2) health literacy and information-seeking capacity; (3) trust in the healthcare system; and (4) social context, including alignment of health views within individuals’ social networks. Given the breadth of potential influences and the use of secondary survey data, this analysis was exploratory in nature, aiming to identify patterns that may inform future hypothesis-driven research. This analysis extends prior work using HINTS data (30–33) and may inform the development of targeted interventions for groups less likely to perceive substantial amounts of health misinformation on social media.

## Materials and methods

HINTS is administered by the National Cancer Institute (34). HINTS 7 data were collected from March to September 2024. Non-institutionalized US adults aged 18 years and older completed surveys via two modes: paper and web. The sampling strategy had a two-stage design: (1) selecting an address from a stratified sample of addresses, and (2) selecting one adult from each sampled household. Participants received a $2 unconditional incentive during the first mailing and were offered a bonus of $10 Amazon electronic gift card to respond on the web. Full methodology of the HINTS 7 (2024) can be found on the HINTS website (35). A total of 7,278 participants completed the HINTS 7 surveys. After including only those with complete data for all variables of interest, 4,741 participants were included for this study’s analyses.

### Perceived frequency of health misinformation on social media

Participants were asked, “How much of the health information that you see on social media do you think is false or misleading?” with response options of a lot, some, a little, and none. For the primary analysis, we compared “a lot/some” vs. “a little/none.” As a sensitivity analysis, we further compared “a lot” vs. all other responses (some, a little, and none).

### Sociodemographic characteristics

We evaluated the following age groups in years: 18–34 (reference), 35–49, 50–64, 65–74, and 75+. Participants were asked “What sex were you assigned at birth, on your original birth certificate?” and had the following response options: male (reference), female, and don’t know. Regarding education, we compared those who were college graduates and those who were not college graduates (reference). Race/ethnicity was evaluated with the following categories: non-Hispanic White (reference), non-Hispanic Black/African American, Hispanic, non-Hispanic Asian, and non-Hispanic other. Participants were asked “Thinking about members of your family living in this household, what is your combined annual income, meaning the total pre-tax income from all sources earned in the past year?” For the analysis of this study, the following categories of household annual income were compared: $0 to $19,999 (reference), $20,000 to $49,999, $50,000 to $99,999, and ≥ $100,000.

### Frequency of internet use

Participants were asked, “About how often do you use the Internet, either on a computer, laptop, smartphone or any other device?” Response options were about once per day, a few times a week, less than once per week, rarely, and never. We categorized responses as the following: “about once per day,” “a few times a week” and “less than once per week” combined, and “rarely” and “never” combined (reference).

### Frequency of social media use

Participants were asked in the past 12 months how often they did the following: (1) visited a social media site, (2) interacted with people who have similar health or medical issues on social media or online forums, and (3) watched a health-related video on a social media site (for example, YouTube). Responses options for each of these three questions were: almost every day, at least once a week, a few times a month, less than once a month, and never. For questions on (1) visiting a social media site and (2) interacting with others who have a similar health issue, we compared “almost every day” to all other responses combined (reference). For the question about (3) frequency of watching a health-related video, we compared those who watched a health-related video (i.e., almost every day to less than once a month) to those who responded “never” (reference).

### Self-rated general health

Participants were asked if they would say their health is excellent, very good, good, fair, or poor. We compared those who responded excellent, very good, and good (reference) to those who responded fair and poor.

### Frequency of going to healthcare provider

Participants were asked, “In the past 12 months, not counting times you went to an emergency room, how many times did you see a doctor, nurse, or other health professional to get care for yourself?” We evaluated responses as the following categories: none (reference), 1–4 times, and 5 or more times.

### Trust in healthcare system

Participants were asked “How much do you trust the healthcare system (for example, hospitals, pharmacies, and other organizations involved in healthcare)?” Response options were a lot, some, a little, and not at all. We compared to those who responded that they had “a lot” with those who responded some, a little, and not at all responses combined (reference).

### Tell whether health information is true/false on social media

Participants were asked how much they agree or disagree that they “find it hard to tell whether health information on social media is true or false.” We compared those who responded strongly agree and somewhat agree with those who responded strongly disagree and somewhat disagree (reference).

### Confidence in filling out medical forms

Participants were asked “How confident are you filling out medical forms by yourself?” Response options were very, somewhat, a little, and not at all. We compared those who responded “very” with all other responses combined (reference). This item is a proxy for limited health literacy skills (36).

### Search skills for health information on internet

Participants were asked how much they agree or disagree with the statement that they have the skills to find the health information they need on the Internet. We compared those who responded strongly agree and somewhat agree with those who responded strongly disagree and somewhat disagree (reference).

### Depression and anxiety

Participants were asked over the past 2 weeks how often they have been bothered by the following problems: (1) little interest or pleasure in doing things, (2) feeling down, depressed, or hopeless, (3) feeling nervous, anxious, or on edge, and (4) not being able to stop or control worrying. Response options for all questions were: nearly every day, more than half the days, several days, and not at all. We assigned a numeric score for each response for each of the 4 questions: nearly every day as 3, more than half the days as 2, and several days as 1, and not at all as 0. The sum of scores for the first two questions was used to evaluate depression. The sum of scores for the last two questions was used to assess anxiety. For each condition, possible scores ranged from 0 to 6. These items are a valid tool to assess depression and anxiety (37).

### Social media network has same views about health

Participants were asked how much they agree or disagree that “most of the people in my social media networks have the same views about health as me.” We compared to those who strongly agree and somewhat agree to those who strongly disagree and somewhat disagree (reference).

### Talk with friends/family about health

Participants were asked “Do you have friends or family members that you talk to about your health?” Response options were yes and no (reference).

### Political viewpoints

Participants were asked, “Thinking about politics these days, how would you describe your own political viewpoint?” We evaluated political viewpoints as liberal (i.e., responses of very liberal, liberal, and somewhat liberal), moderate, and conservative (i.e., responses of very conservative, conservative, or somewhat conservative).

### Belonging with own ethnic group

Participants were asked how much they agree or disagree that they “have a strong sense of belonging to my own ethnic group.” Response options were strongly agree, agree, neither agree nor disagree, disagree, and strongly disagree. We evaluated responses as the following: strongly agree/agree, neither agree nor disagree (reference), and strongly disagree/disagree.

### Data analysis

Logistic regression models were used to determine factors associated with perceiving a lot/some health misinformation. As a sensitivity analysis, additional models were created to determine factors associated with perceiving a lot of health misinformation. Factors with an overall *p*-value of less than 0.10 in univariable models were considered in multivariable models. Backward elimination was used to arrive at a parsimonious model given the large number of candidate predictors and the exploratory nature of this analysis. Independent variables were sequentially removed until all remaining variables were statistically significant at *p*-value < 0.05. Analyses were conducted in R version 4.5.1 with the following packages: haven, dplyr, survey, srvyr, and broom (38). A survey design object accounting for jackknife weighting and replicate weights was created with the function “as_survey_rep” (38). Logistic regression models using this survey design object were created using the function “svyglm” (38). Weighted percentages, adjusted odds ratios (aOR), 95% confidence intervals (95% CI), and *p*-values are reported.

## Results

### Characteristics of the analytical sample

Supplementary Table 1 summarizes the characteristics of the 4,741 included participants, including both weighted and unweighted percentages. Based on weighted estimates, nearly 60% of participants were between ages 18 and 49, with slightly more males than females (51%), a majority of participants were non-college graduates, and representation across race/ethnicity and income levels. Most participants (84%) reported excellent, very good, or good general health.

### Primary analysis comparing a lot/some vs. a little/none perceived health misinformation

Weighted row percentages and univariable logistic regression models for perceiving a lot/some health misinformation on social media are in Table 1. Exactly 81.6% (weighted percentage) perceived lot/some health misinformation and 18.4% perceived a little/no health misinformation on social media. The multivariable logistic regression model results for perceiving a lot/some health misinformation are in Table 2. Compared to the 18–34 years age group, participants who were 50–64 years (OR = 1.67 [95% CI: 1.15, 2.42]) and 65–74 years (OR = 1.89 [95% CI: 1.25, 2.86]) had greater odds of perceiving a lot/some misinformation. College graduates (OR = 1.53 [95% CI: 1.08, 2.18]) had greater odds of perceiving a lot/some misinformation compared to those who were non-college graduates. Black/African American (OR = 0.54 [95% CI: 0.36, 0.81]) and Hispanic (OR = 0.49 [95% CI: 0.34, 0.69]) participants had lower odds of perceiving a lot/some misinformation compared to White participants. Those with an annual household income of $50,000–$99,999 (OR = 1.63 [95% CI: 1.02, 2.61]) and $100,000 or more (OR = 1.75 [95% CI: 1.03, 2.98]) had greater odds of perceiving a lot/some misinformation than those in the $0–$19,999 range. Participants who agreed (OR = 0.64 [95% CI: 0.48, 0.84]) that people in their social media network have the same views on health as them had lower odds of perceiving a lot/some misinformation compared to those who disagreed.

**TABLE 1:** Weighted percentages and univariable logistic regression model results for perceiving “a Lot/Some” health misinformation on social media (*n* = 4,741).

	“How much of the health information that you see on social media do you think is false or misleading?”			
Characteristics	A lot/some % (SE)	A little/none % (SE)	Unadjusted OR [95% CI]^‡^	*P*-value
Number of participants (weighted %)	3,925 (81.6%)	816 (18.4%)		
Age group				Overall < 0.001
18–34	74.8 (2.3)	25.2 (2.3)	Reference	–
35–49	82.7 (1.7)	17.3 (1.7)	1.61 [1.13, 2.31]	0.010
50–64	86.3 (1.6)	13.7 (1.6)	2.12 [1.47, 3.06]	< 0.001
65–74	87.2 (1.6)	12.8 (1.6)	2.30 [1.59, 3.33]	< 0.001
75+	77.5 (6.1)	22.5 (6.1)	1.16 [0.51, 2.68]	0.714
Birth sex				Overall = 0.072
Female	83.4 (1.2)	16.6 (1.2)	1.27 [1.00, 1.61]	0.052
Male	79.9 (1.4)	20.1 (1.4)	Reference	–
Don’t know	62.7 (21.2)	37.3 (21.2)	0.42 [0.05, 3.46]	0.413
Education				
Not college graduate	78.2 (1.4)	21.8 (1.4)	Reference	–
College graduate	87.4 (1.2)	12.6 (1.2)	1.94 [1.46, 2.58]	< 0.001
Race/ethnicity				Overall < 0.001
Non-Hispanic White	86.0 (1.0)	14.0 (1.0)	Reference	–
Non-Hispanic Black or African American	75.5 (2.9)	24.5 (2.9)	0.50 [0.35, 0.73]	< 0.001
Hispanic	71.1 (2.8)	28.9 (2.8)	0.40 [0.29, 0.57]	< 0.001
Non-Hispanic Asian	80.3 (6.8)	19.7 (6.8)	0.66 [0.26, 1.71]	0.389
Non-Hispanic other	78.7 (4.4)	21.3 (4.4)	0.60 [0.34, 1.08]	0.089
Income				Overall < 0.001
0 to $19,999	71.6 (3.7)	28.4 (3.7)	Reference	–
20,000 to $49,999	74.9 (2.3)	25.1 (2.3)	1.18 [0.74, 1.89]	0.472
50,000 to $99,999	83.9 (1.5)	16.1 (1.5)	2.07 [1.31, 3.29]	0.003
≥ $100,000	87.3 (1.5)	12.7 (1.5)	2.73 [1.69, 4.43]	< 0.001
Frequency of internet use				Overall = 0.021
More than once a day	82.6 (0.9)	17.4 (0.9)	1.69 [0.38, 7.51]	0.483
About once per day and less than once per week	70.3 (4.9)	29.7 (4.9)	0.85 [0.18, 4.07]	0.828
Rarely and never	73.8 (12.6)	26.2 (12.6)	Reference	–
Frequency of visiting social media site				
Almost every day	81.9 (1.1)	18.1 (1.1)	1.10 [0.85, 1.41]	0.460
Not almost every day (at least once a week to never)	80.5 (1.6)	19.5 (1.6)	Reference	–
Interact with people with similar health issue on social media				
Almost every day	67.3 (9.3)	32.7 (9.3)	0.46 [0.19, 1.13]	0.087
Not almost every day (at least once a week to never)	81.8 (1.0)	18.2 (1.0)	Reference	–
Watching health-related video on social media				
Watched (almost every day to less than once a month)	81.9 (1.0)	18.1 (1.0)	1.09 [0.85, 1.40]	0.483
Never	80.5 (1.8)	19.5 (1.8)	Reference	–
General health				
Fair and poor	80.4 (2.3)	19.6 (2.3)	0.91 [0.66, 1.27]	0.580
Excellent, very good, and good	81.8 (1.0)	18.2 (1.0)	Reference	–
Frequency of visiting health professional				Overall = 0.003
None	75.2 (3.0)	24.8 (3.0)	Reference	–
1 to 4 times	80.7 (1.4)	19.3 (1.4)	1.38 [0.95, 2.00]	0.086
≥ 5 times	86.4 (1.3)	13.6 (1.3)	2.09 [1.37, 3.20]	0.001
Trust in healthcare system				
A lot	82.4 (1.9)	17.6 (1.9)	1.09 [0.78, 1.51]	0.618
Not a lot (some, a little, and not at all)	81.1 (1.2)	18.9 (1.2)	Reference	–
Hard to tell whether health information on social media is true or false				
Strongly/somewhat agree	82.1 (1.2)	17.9 (1.2)	1.10 [0.84, 1.45]	0.490
Strongly/somewhat disagree	80.6 (1.7)	19.4 (1.7)	Reference	–
Confidence in filling out medical form				
Very	84.6 (1.2)	15.4 (1.2)	1.79 [1.27, 2.52]	0.001
Not very (somewhat, a little, and not at all)	75.3 (2.3)	24.7 (2.3)	Reference	–
Search skills to find health information needed on the internet				
Strongly/somewhat agree	81.8 (0.9)	18.2 (0.9)	1.24 [0.72, 2.15]	0.432
Strongly/somewhat disagree	78.4 (4.4)	21.6 (4.4)	Reference	–
Score on depression scale (median [IQR])	0 [0, 2]	1 [0, 2]	0.92 [0.84, 1.00]	0.051
Score on anxiety scale (median [IQR])	0 [0, 2]	0 [0, 2]	0.96 [0.89, 1.04]	0.364
People in my social media network has same views on health as me				
Strongly/somewhat agree	78.8 (1.4)	21.2 (1.4)	0.70 [0.54, 0.91]	0.008
Strongly/somewhat disagree	84.1 (1.3)	15.9 (1.3)	Reference	–
Have friends or family to talk about health				
Yes	82.1 (0.9)	17.9 (0.9)	1.20 [0.86, 1.69]	0.276
No	79.2 (2.7)	20.8 (2.7)	Reference	–
Political viewpoint				Overall = 0.140
Conservative	81.0 (1.8)	19.0 (1.8)	1.11 [0.79, 1.57]	0.548
Moderate	79.4 (1.7)	20.6 (1.7)	Reference	–
Liberal	84.9 (1.9)	15.1 (1.9)	1.46 [1.00, 2.13]	0.052
Strong sense of belonging to my own ethnic group				Overall = 0.060
Strongly agree and agree	79.9 (1.5)	20.1 (1.5)	0.85 [0.63, 1.15]	0.282
Neither agree nor disagree	82.4 (1.5)	17.6 (1.5)	Reference	–
Strongly disagree and disagree	86.7 (2.0)	13.3 (2.0)	1.39 [0.97, 2.01]	0.076

*Row percentages are weighted.

† SE is weighted standard error.

‡ OR is odds ratio and 95% CI is 95% confidence interval.

**TABLE 2:** Multivariable logistic regression results for perceiving “a Lot/Some” health misinformation on social media (*n* = 4,741).

	“How much of the health information that you see on social media do you think is false or misleading?”	
Characteristics	Adjusted OR [95% CI]*	*P*-value
Age group		Overall = 0.016
18–34	Reference	–
35–49	1.28 [0.87, 1.86]	0.202
50–64	1.67 [1.15, 2.42]	< 0.001
65–74	1.89 [1.25, 2.86]	0.004
75+	0.91 [0.38, 2.17]	0.831
Education		
Not college graduate	Reference	–
College graduate	1.53 [1.08, 2.18]	0.019
Race/ethnicity		Overall = 0.001
Non-Hispanic White	Reference	–
Non-Hispanic Black or African American	0.54 [0.36, 0.81]	0.004
Hispanic	0.49 [0.34, 0.69]	< 0.001
Non-Hispanic Asian	0.72 [0.28, 1.82]	0.474
Non-Hispanic other	0.69 [0.37, 1.28]	0.229
Income		Overall = 0.008
$0 to $19,999	Reference	–
$20,000 to $49,999	1.06 [0.65, 1.74]	0.806
$50,000 to $99,999	1.63 [1.02, 2.61]	0.044
≥ $100,000	1.75 [1.03, 2.98]	0.039
People in my social media network has same views on health as me		
Strongly/somewhat agree	0.64 [0.48, 0.84]	0.002
Strongly/somewhat disagree	Reference	–

*OR is odds ratio and 95% CI is 95% confidence interval.

### Sensitivity analyses comparing “a Lot” to all other categories of perceived health misinformation

Weighted row percentages and univariable logistic regression models for perceiving a lot of health misinformation on social media are in Table 3. 36.4% (weighted percentage) perceived lot and 63.6% perceived some/a little/no health misinformation on social media. The multivariable logistic regression model results for perceiving a lot of health misinformation results are in Table 4. Participants who were 50–64 years (OR = 1.54 [95% CI: 1.12, 2.12]) had greater odds of perceiving a lot of misinformation than those who were 18–34 years. College graduates (OR = 1.45 [95% CI: 1.12, 1.88]) had greater odds of perceiving a lot of misinformation compared to those who were non-college graduates. Black/African American (OR = 0.51 [0.35, 0.75]) and Hispanic (OR = 0.56 [0.42, 0.76]) participants had lower odds of perceiving a lot of misinformation compared to White participants. Participants who agreed (OR = 0.64 [95% CI: 0.48, 0.84]) that people in their social media network have the same views on health as themselves had lower odds of perceiving a lot of misinformation compared to those who disagreed. Having a lot of trust in the healthcare system (OR = 0.70 [95% CI: 0.54, 0.93]), when compared to those who did not respond “a lot,” was linked to lower odds of perceiving a lot of misinformation. Individuals that agreed to having the skills needed to find health information they need on the internet (OR = 0.60 [95% CI: 0.41, 0.87]) had lower odds of perceiving a lot of health misinformation on social media. Participants who reported that they disagreed (OR = 1.60 [1.12, 2.30]) with having a strong sense of belonging to one’s own ethnic group had greater odds of perceiving a lot of misinformation than those who neither agreed nor disagreed.

**TABLE 3:** Weighted percentages and univariable logistic regression model results for perceiving “a Lot” of health misinformation on social media (*n* = 4,741).

	“How much of the health information that you see on social media do you think is false or misleading?”			
Characteristics	A lot % (SE)^†^	Some/a little/none % (SE)	Unadjusted OR [95% CI]^‡^	*P*-value
Number of participants (weighted %)	1,734 (36.4%)	3,007 (63.6%)		
Age group				Overall = 0.030
18–34	30.9 (2.8)	69.1 (2.8)	Reference	–
35–49	37.4 (2.5)	62.6 (2.5)	1.33 [0.94, 1.90]	0.108
50–64	42.2 (2.6)	57.8 (2.6)	1.63 [1.17, 2.26]	0.005
65–74	36.9 (2.5)	63.1 (2.5)	1.31 [0.93, 1.84]	0.125
75+	30.9 (3.5)	69.1 (3.5)	1.00 [0.66, 1.52]	0.993
Birth sex				Overall = 0.357
Female	38.1 (1.8)	61.9 (1.8)	1.16 [0.93, 1.44]	0.192
Male	34.7 (1.8)	65.3 (1.8)	Reference	–
Don’t know	53.1 (19.5)	46.9 (19.5)	2.13 [0.40, 11.2]	0.366
Education				
Not college graduate	33.5 (1.8)	66.5 (1.8)	Reference	–
College graduate	41.7 (1.6)	58.3 (1.6)	1.42 [1.14, 1.76]	0.002
Race/ethnicity				Overall < 0.001
Non-Hispanic White	40.8 (1.7)	59.2 (1.7)	Reference	–
Non-Hispanic Black or African American	25.7 (3.0)	74.3 (3.0)	0.50 [0.35, 0.71]	< 0.001
Hispanic	26.7 (2.4)	73.3 (2.4)	0.53 [0.39, 0.71]	< 0.001
Non-Hispanic Asian	29.2 (5.8)	70.8 (5.8)	0.60 [0.34, 1.05]	0.071
Non-Hispanic other	47.4 (5.4)	52.6 (5.4)	1.30 [0.83, 2.06]	0.249
Income				Overall = 0.003
0 to $19,999	34.1 (4.0)	65.9 (4.0)	Reference	–
20,000 to $49,999	29.4 (2.6)	70.6 (2.6)	0.81 [0.54, 1.19]	0.274
50,000 to $99,999	35.8 (2.5)	64.2 (2.5)	1.08 [0.72, 1.62]	0.713
≥ $100,000	42.0 (1.9)	58.0 (1.9)	1.40 [0.95, 2.05]	0.084
Frequency of internet use				Overall = 0.18
More than once per day	37.1 (1.3)	62.9 (1.3)	0.64 [0.25, 1.63]	0.342
About once per day and a few times a day	29.7 (4.0)	70.3 (4.0)	0.46 [0.16, 1.32]	0.146
Rarely and never	47.9 (11.3)	52.1 (11.3)	Reference	–
Frequency of visiting social media site				
Almost every day	35.1 (1.4)	64.9 (1.4)	0.80 [0.64, 1.00]	0.051
Not almost every day (at least once a week to never)	40.4 (2.3)	59.6 (2.3)	Reference	–
Interact with people with similar health issue on social media				
Almost every day	24.4 (7.5)	75.6 (7.5)	0.56 [0.25, 1.25]	0.153
Not almost every day (at least once a week to never)	36.7 (1.2)	63.3 (1.2)	Reference	–
Watching health-related video on social media				
Watched (almost every day to less than once a month)	34.7 (1.6)	65.3 (1.6)	0.73 [0.56, 0.94]	0.016
Never	42.3 (2.2)	57.5 (2.2)	Reference	–
General health				
Fair and poor	36.4 (3.1)	63.6 (3.1)	1.00 [0.75, 1.34]	0.999
Excellent, very good, and good	36.4 (1.3)	63.6 (1.34)	Reference	–
Frequency of visiting health professional				Overall = 0.048
None	35.9 (4.6)	63.1 (4.6)	Reference	–
1 to 4 times	34.2 (1.4)	65.8 (1.4)	0.93 [0.62, 1.40]	0.717
≥ 5 times	42.0 (2.7)	58.0 (2.7)	1.29 [0.81, 2.06]	0.271
Trust in healthcare system				
A lot	31.1 (2.0)	68.9 (2.0)	0.70 [0.55, 0.90]	0.006
Not a lot (some, a little, and not at all)	39.1 (1.7)	60.9 (1.7)	Reference	–
Hard to tell whether health information on social media is true or false				
Strongly/somewhat agree	38.4 (1.6)	61.6 (1.6)	1.27 [1.00, 1.61]	0.046
Strongly/somewhat disagree	32.8 (2.1)	67.2 (2.1)	Reference	–
Confidence in filling out medical form				
Very	38.0 (1.2)	62.0 (1.2)	1.24 [0.95, 1.61]	0.120
Not very (somewhat, a little, and not at all)	33.2 (2.8)	66.8 (2.8)	Reference	–
Search skills to find health information needed on the internet				
Strongly/somewhat agree	35.6 (1.4)	64.4 (1.4)	0.66 [0.46, 0.95]	0.026
Strongly/somewhat disagree	45.6 (3.9)	54.4 (3.9)	Reference	–
Score on depression scale (median [IQR])	0 [0, 2]	0 [0, 2]	1.04 [0.94, 1.14]	0.473
Score on anxiety scale (median [IQR])	1 [0, 2]	1 [0, 2]	1.05 [0.97, 1.13]	0.201
People in my social media network has same views on health as me				
Strongly/somewhat agree	31.8 (1.4)	68.2 (1.4)	0.68 [0.57, 0.81]	< 0.001
Strongly/somewhat disagree	40.8 (1.7)	59.2 (1.7)	Reference	–
Have friends or family to talk about health				
Yes	36.6 (1.3)	63.4 (1.3)	1.03 [0.76, 1.40]	0.823
No	35.8 (3.4)	64.2 (3.4)	Reference	–
Political viewpoint				Overall = 0.052
Conservative	39.6 (2.0)	60.4 (2.0)	1.31 [1.05, 1.63]	0.016
Moderate	33.4 (2.0)	66.6 (2.0)	Reference	–
Liberal	37.0 (2.0)	63.0 (2.0)	1.17 [0.91, 1.51]	0.213
Strong sense of belonging to my own ethnic group				Overall < 0.001
Strongly agree and agree	32.8 (1.8)	67.2 (1.8)	0.80 [0.63, 1.10]	0.077
Neither agree nor disagree	37.9 (2.0)	62.1 (2.0)	Reference	–
Strongly disagree and disagree	50.0 (3.8)	50.0 (3.8)	1.64 [1.16, 2.32]	0.007

*Row percentages are weighted.

† SE is the weighted standard error.

‡ OR is odds ratio and 95% CI is 95% confidence interval.

**TABLE 4:** Multivariable logistic regression results for perceiving “A Lot” of health misinformation on social media (*n* = 4,741).

	“How much of the health information that you see on social media do you think is false or misleading?”	
Characteristics	Adjusted OR [95% CI]*	*P*-value
Age group		Overall = 0.033
18–34	Reference	–
35–49	1.24 [0.87, 1.77]	0.230
50–64	1.54 [1.12, 2.12]	0.009
65–74	1.27 [0.90, 1.79]	0.166
75+	0.89 [0.55, 1.43]	0.609
Education		
Not college graduate	Reference	–
College graduate	1.45 [1.12, 1.88]	0.006
Race/ethnicity		Overall < 0.001
Non-Hispanic White	Reference	–
Non-Hispanic Black or African American	0.51 [0.35, 0.75]	0.001
Hispanic	0.56 [0.42, 0.76]	< 0.001
Non-Hispanic Asian	0.62 [0.34, 1.13]	0.118
Non-Hispanic other	1.31 [0.82, 2.11]	0.250
Trust in healthcare system		
A lot	0.70 [0.54, 0.93]	0.014
Not a lot (some, a little, and not at all)	Reference	–
Search skills to find health information needed on the internet		
Strongly/somewhat agree	0.60 [0.41, 0.87]	0.009
Strongly/somewhat disagree	Reference	–
People in my social media network has same views on health as me		
Strongly/somewhat agree	0.66 [0.55, 0.79]	< 0.001
Strongly/somewhat disagree	Reference	–
Strong sense of belonging to my own ethnic group		Overall = 0.018
Strongly agree and agree	0.94 [0.72, 1.21]	0.604
Neither agree nor disagree	Reference	–
Strongly disagree and disagree	1.60 [1.12, 2.30]	0.012

*OR is odds ratio and 95% CI is 95% confidence interval.

## Discussion

Using 2024 nationally representative survey data on US adults, an estimated 81.6% had perceived a lot/some health misinformation and 18.4% perceived a little/no false or misleading health information (i.e., health misinformation) when using social media. In the primary analysis, age group, education, race/ethnicity, income, and whether one’s social media network had the same views on health were associated with perceiving a lot/some health misinformation. In the sensitivity analysis comparing those who believed there was “a lot” of health misinformation to all other responses, 36.4% perceived a lot of health misinformation and 63.6% perceived there was some, a little, or no health misinformation. Age group, education, race/ethnicity, and whether those in a participants’ social media network had the same views on health were associated with perceiving a lot of health misinformation on social media. Trust in the healthcare system, search skills to find needed health information on the internet, and a strong sense of belonging to one’s own ethnic group were only significant in the sensitivity analysis with perceiving a lot of misinformation. Our findings are broadly consistent with prior analyses of HINTS data, which have reported high perceived exposure to health misinformation and similar patterns by race/ethnicity and income (30–33). Differences in associations with age and education may reflect variation in the multivariable model specification and variable categorization across studies.

### Social media network has same views on health

Social media users with a network of similarly minded individuals could lead to users frequently coming across health information that aligns with their previously held views and perceiving such information as correct (i.e., confirmation bias) (39, 40). Social media users may search through certain tags on social media sites (e.g., Twitter/X) to find health information that aligns with their previous beliefs, such as finding posts with their viewpoint on the COVID-19 vaccine (3).

### Race/ethnicity and ethnic belonging

In Fareed et al.’s (41) analysis of pooled HINTS data from 2007 to 2017, Black and Hispanic participants had greater odds of reporting that they had a lot of trust in the internet for health information than White participants. It is possible that Black and Hispanic individuals trust and purposely seek health information on social media, which leads to less perceived health misinformation. A unique finding of this study that warrants further investigation is how those who disagreed to having a strong sense of ethnic belonging were more likely to perceive a lot of health misinformation. How much individuals identify with their ethnic group could shape what information is viewed as factual and biased toward one’s own ethnic group (42), which may affect perceived health misinformation on social media.

### Age group

Younger adults (aged 18 to 29 years) were more likely to report using Instagram, TikTok, and Reddit in general when compared to older adults to a Pew Research Center survey in 2025 (12). A KFF Tracking Poll in 2025 found that 18–29 year olds more regularly got health information and advice from social media influencers (23%), and a greater percentage reported that most/some of the health information on TikTok (54%), YouTube (47%), and Instagram (38%) were trustworthy, when compared to older age groups (43). Thus, younger adults may perceive less health misinformation due to seeking health information from more trusted social media sites and influencers.

### Trust in healthcare system and search skills finding health information on internet

Healthcare providers often provide better quality and education in their videos and posts than other social media users (44–46). Those with a lot of trust in the healthcare system may seek the advice of health professionals and organizations on these social media sites leading to less perceived misinformation on social media. Another possibility is those with higher trust may rely more on consultations with healthcare providers and websites for health information rather than social media for health information. Similarly, those who are confident in their skills to find health information needed on the internet may also seek out sources they believe to be reputable on social media or use sources they trust outside of social media for health information.

### College education and income level

College graduates perceiving higher amounts of health misinformation could be due to higher health literacy and general knowledge of scientific information (47–49) which could aid in identifying health misinformation. Differences in perceptions of health misinformation among annual household income ranges may be attributed to frequency of social media use and social media sites more commonly used by certain groups. According to a 2023 survey, adults with higher household incomes (i.e., greater than $100,000) had greater rates of using Twitter/X than those belonging to lower income ranges (i.e., less than $30,000, $30,000–$69,999, and $70,000–$99,999) (50).

### Strengths and limitations

Strengths include a large, nationally representative sample, inclusion of multiple potential correlates, and the use of sensitivity analyses. However, some limitations must be noted. This study cannot distinguish between exposure to misinformation and individual skepticism toward health information. Given the cross-sectional design, reverse causality cannot be ruled out. In addition, the HINTS 2024 survey did not ask participants which social media sites participants used, limiting conclusions about differences across platforms.

### Future directions

Further research is needed to understand why individuals who report lower levels of ethnic belonging perceive greater amounts of health misinformation on social media. Future studies should also further examine how accurately individuals identify false or misleading health information, particularly how this ability varies across sociodemographic groups and in different information environments. Given differences across social media platforms, researchers should assess platform-specific variation in perceived misinformation and how trust in health institutions moderates these perceptions. Finally, while interventions such as social media campaigns, educational efforts, and warning labels have been developed (51–53), further research is needed to determine how these strategies influence perceptions of misinformation, particularly when content aligns or misaligns with prior beliefs.

## Conclusion

In this nationally representative analysis of US adults, perceptions of health misinformation on social media varied substantially across sociodemographic groups, levels of trust in the healthcare system, and the degree of alignment in individuals’ social media networks. Older adults, college graduates, and those with higher incomes were more likely to perceive higher amounts of misinformation, whereas Black and Hispanic adults and those embedded in like-minded social media networks were less likely to do so. Sensitivity analyses further highlighted the roles of trust in health systems, online search skills, and ethnic identity belonging with perceptions of health misinformation. These patterns suggest that perceptions of misinformation are shaped not only by individual characteristics but also by social context and informational environments. Efforts to design targeted public health messaging and strengthen media literacy may benefit from accounting for these group differences and the mechanisms that underlie them.

## Data Availability

Publicly available datasets were analyzed in this study. This data can be found here: https://hints.cancer.gov/. Data from the HINTS 7 (2024) were analyzed.
