# Supplementary material for: Perceptions of health misinformation on social media: patterns and predictors
Source: Front Med (Lausanne). 2026 May 15;13:1770275. doi: 10.3389/fmed.2026.1770275 (PMC13218912; doi:10.3389/fmed.2026.1770275)
Supplement: Supplementary file 1 [file Table_1.docx]

**Supplementary Table 1: Sociodemographic Characteristics of Analytical Sample (n = 4741)**

|  | Number of Participants | Unweighted % | Weighted % |
| --- | --- | --- | --- |
| **Age Group** |  |  |  |
| 18-34 | 978 | 20.6 | 29.2 |
| 35-49 | 1181 | 24.9 | 29.7 |
| 50-64 | 1312 | 27.7 | 26.6 |
| 65-74 | 856 | 18.1 | 10.0 |
| 75+ | 414 | 8.7 | 4.5 |
|  |  |  |  |
| **Birth Sex** |  |  |  |
| Female | 1856 | 39.1 | 48.8 |
| Male | 2862 | 60.4 | 50.8 |
| Don’t Know | 23 | 0.5 | 0.5 |
|  |  |  |  |
| **Education** |  |  |  |
| Not college graduate | 2258 | 47.6 | 63.6 |
| College graduate | 2483 | 52.4 | 36.4 |
|  |  |  |  |
| **Race/Ethnicity** |  |  |  |
| Non-Hispanic White | 2602 | 54.9 | 60.8 |
| Non-Hispanic Black  or African American | 674 | 14.2 | 10.5 |
| Hispanic | 989 | 20.9 | 17.6 |
| Non-Hispanic Asian | 280 | 5.9 | 5.9 |
| Non-Hispanic Other | 195 | 4.1 | 5.3 |
|  |  |  |  |
| **Income** |  |  |  |
| $0 to $19,999 | 663 | 14.0 | 13.4 |
| $20,000 to $49,999 | 1096 | 23.0 | 21.7 |
| $50,000 to $99,999 | 1432 | 30.2 | 28.6 |
| ≥ $100,000 | 1550 | 32.7 | 36.3 |
|  |  |  |  |
| **General Health** |  |  |  |
| Fair and poor | 828 | 17.5 | 16.6 |
| Excellent, very good, and good | 3913 | 82.5 | 84.4 |
